# Utilisation of dental services for people with special health care needs in Australia

**DOI:** 10.1186/s12903-020-01354-6

**Published:** 2020-12-11

**Authors:** Mathew Albert Wei Ting Lim, Sharon Andrea Corinne Liberali, Gelsomina Lucia Borromeo

**Affiliations:** 1 Melbourne Dental School, University of Melbourne, Carlton, Australia; 2 Dental Services, Alfred Health, Melbourne, Australia; 3 Maxillofacial Surgery and Dental Clinic, Royal Melbourne Hospital, Parkville, Australia; 4 Adelaide Dental School, The University of Adelaide, Adelaide, Australia; 5 Special Needs Unit, Adelaide Dental Hospital, Adelaide, Australia

**Keywords:** Delivery of health care, Dental care for aged, Dental care for chronically ill, Dental care for disabled, Health services accessibility

## Abstract

**Background:**

To explore the profile of patients and treatment delivered at specialist referral centres for individuals with special needs.

**Methods:**

A cross-sectional audit was conducted of the health records of all patients with appointments at two of Australia’s largest referral centres for patients with special needs, the Integrated Special Needs Department at the Royal Dental Hospital of Melbourne and the Special Needs Unit at the Adelaide Dental Hospital, for the month of August 2015.

**Results:**

The profile of patients treated at these specialist units demonstrates the diversity of individuals with additional health care needs that general dentists feel require specialised oral health care. The Adelaide-based clinic had a greater proportion of complex medical patients in comparison to those treated in Melbourne who were more likely to have a disability or psychiatric condition and were less likely to be able to self-consent for treatment. Interestingly, despite similar workforce personnel numbers, there were approximately twice as many appointments at the Special Needs Unit in Adelaide than the Integrated Special Needs Department in Melbourne during the study period which may have reflected differences in workforce composition with a greater use of dental auxiliaries at the Adelaide clinic.

**Conclusions:**

The results of this study provide an initial profile of patients with special needs referred for specialist care in Australia. However, the differences in patient profiles between the two units require further investigation into the possible influence of service provision models and barriers to access of care for individuals with special needs and to ensure equitable access to health care.

## Background

Individuals with special needs, ranging from those with disabilities to those with complex health issues, commonly report experiencing problems with accessing routine dental care [1–3]. Worryingly, despite increasing advocacy for these patient groups, improvements to university training programs, and increasing information about the links between oral and systemic health, these individuals continue to experience problems with accessing dental care largely due to the reluctance of many oral health professionals to treat them [2, 4, 5].

In order to address this ongoing issue, Australia and New Zealand were amongst the first countries to establish special needs dentistry as a registrable dental specialty and, in doing so, recognise the growing group of individuals within our adult population with additional health care needs, and how this can impact on their oral health or ability to access health care [6, 7]. Previously, many of these individuals were managed by paediatric dental specialists who continued to provide care to children with special needs [8]. Consequently, special needs dentistry has evolved to be defined as a speciality that “supports the oral health care needs of people with an intellectual disability, medical, physical or psychiatric conditions that require special methods or techniques to prevent or treat oral health problems, or where such conditions necessitate special dental treatment plans” [9].

Despite this positive move forward, special needs dentistry remains a relatively new dental specialty and little is known about the utilisation of referral-based services provided by the specialty in countries like Australia, New Zealand, or the United Kingdom [10–12]. This information is vital to ensuring adequate access to dental services for these individuals as well as informing other countries who may be considering whether the recognition of such a specialty is necessary. In Australia, government-funded tertiary dental facilities in several major cities have established referral-based clinics specialised in and dedicated solely to the management of patients with special needs. The five major referral centres across the country include specialist departments at the Royal Dental Hospital of Melbourne in Melbourne, Victoria, the Adelaide Dental Hospital in Adelaide, South Australia, the Sydney Dental Hospital and Westmead Oral Health Centre in Sydney, New South Wales, and the University of Queensland Oral Health Centre in Brisbane, Queensland. Although the organisation of community dental services and hospital dental clinics vary between states, the specialist workforce at these specialised clinics provides support to their own staff and the wider public dental system in managing the oral health needs of the eligible population of individuals with special needs.

This study will examine the patients treated and services provided by two of the largest and most established specialist units in Australia: the Integrated Special Needs Department (ISND) at the Royal Dental Hospital of Melbourne, Victoria and the Special Needs Unit (SNU) at the Adelaide Dental Hospital, South Australia. In addition to establishing a valuable profile of the types of patients with special needs referred for specialist care in this country, comparison between these two units with different service models, may assist in developing a greater understanding of how differences in service provision may influence access to dental care and the nature of dental care provided for individuals with special needs.

## Methods

A cross-sectional review was conducted of all patient appointments at referral-based specialist units for patients with special needs at the Royal Dental Hospital of Melbourne and Adelaide Dental Hospital between August 1, 2015 and August 31, 2015. Both are the major referral centres for the oral health care of eligible patients with additional health care needs in their respective states. At the Royal Dental Hospital of Melbourne appointments included those provided by the Integrated Special Needs Department (ISND), Domiciliary Service, and patients treated on dedicated ISND general anaesthetic lists at the Day Surgery Unit located at the Royal Dental Hospital of Melbourne. At the Special Needs Unit (SNU) in Adelaide, appointments included those provided at the specialist unit at the Adelaide Dental Hospital (ADH) as well as dental clinics at The Queen Elizabeth Hospital (TQEH), Highgate Park, and the Modbury GP Plus Superclinic.

The patients treated during the audit period were identified from electronic appointment books. Patient records were reviewed for demographic information (gender, date of birth), ability to consent, eligibility for public dental care, reason for referral, medical history, and treatment received.

Within the Australian setting, individuals are generally only able to access government-funded public dental services based on level of socio-economic disadvantage except in special circumstances which can vary between systems. For example, individuals in South Australia who are receiving treatment at certain public hospitals may be able to access public dental care only whilst they remain inpatients. Eligibility for public dental care is otherwise usually determined by whether an individual holds an Australian government-issued concession health care card or pensioner card which were the criteria for eligibility used in this study.

The audit of clinical records was conducted by a single investigator with data recorded using a standardised data collection sheet developed by the research team. Medical conditions and medications were recorded using the World Health Organization International Classification of Disease 10 (ICD-10) and the Anatomical Therapeutic Chemical and Defined Daily Dose (ATC/DDD) classifications [13, 14]. All medical conditions, not just the primary reason for referral, were recorded. SPSS Statistics Version 23 (IBM Inc, Armonk NY, USA) was used for data analysis. Although this analysis was primarily descriptive, statistical tests, including chi-square tests and confidence intervals, were used where required with statistical significance reported at the level of p < 0.05.

As all data obtained from patient records was deidentified, and due to the impractical nature of contacting each individual patient, it was deemed that consent for access to individual records was not required by the relevant human research ethics committees. Ethics approval for this project was obtained from the Melbourne Dental School Human Ethics Advisory Group and University of Melbourne Human Research Ethics Committee (Ethics ID 1544156), Dental Health Services Victoria (DHSV) (ID 297), SA Health Human Research Ethics Committee (HREC Ref No. HREC/15/SAH/141), and Central Adelaide Local Health Network/South Australian Dental Service Evaluation and Research Unit (LNR SSA Ref No. AU/16/4353215).

## Results

During the study period, 1908 appointments were reviewed across the two centres with approximately two-thirds of these appointments (64.6%) affiliated with the Special Needs Unit (SNU) in Adelaide. A full breakdown of the number of appointments by clinic is provided in Table 1.

**Table 1:** Number of appointments and patients across the Royal Dental Hospital of Melbourne (Melbourne, Victoria) and the Special Needs Unit (Adelaide, South Australia) between August 1, 2015 and August 31, 2015 divided by clinic and type of oral health professional

	Royal Dental Hospital of Melbourne			Special Needs Unit, Adelaide			
	ISND	Dom	Total RDHM	ADH	TQEH	Other	Total SNU
Specialist^a^	103 (23.5)	13 (5.5)	116	344 (34.6)	0 (0.0)	30 (63.8)	374
Training specialist^a^	0 (0.0)	14 (5.9)	14	36 (3.6)	1 (0.5)	3 (6.4)	40
General dentist	229 (52.2)	137 (58.1)	366	478 (48.1)	191 (99.0)	4 (8.5)	673
Hygienist	7 (1.6)	0 (0.0)	7	102 (10.3)	0 (0.0)	10 (21.3)	112
Prosthetist	2 (0.5)	69 (29.2)	71	0 (0.0)	0 (0.0)	0 (0.0)	0
Dental student	0 (0.0)	0 (0.0)	0	32 (3.2)	0 (0.0)	0 (0.0)	32
Other specialty	20 (4.6)	1 (0.4)	21	0 (0.0)	0 (0.0)	0 (0.0)	0
Cancelled appointments	78 (17.8)	2 (0.9)	80	1 (0.1)	1 (0.5)	0 (0.0)	2
Total appointments	439	236	675	993	193	47	1233
Patients treated	368	213	579	688	111	45	844

Some patients had more than one appointment scheduled during the study period

*ISND* Integrated Special Needs Department + Day Surgery Unit, *Dom* Domiciliary dental services, *RDHM* Royal Dental Hospital of Melbourne, *ADH* Adelaide Dental Hospital, *TQEH* The Queen Elizabeth Hospital, *Other* Highgate Park and Modbury GP Plus Superclinic, *SNU* Special Needs Unit

^a^Refers to specialists and training specialists in special needs dentistry

During the study period, repeat appointments for the same patient represented approximately one quarter of appointments in the Victorian group (n = 171, 25.3%) and almost half of appointments in the South Australian cohort (n = 570, 46.2%). 14.4% (n = 97) of appointments at the RDHM in Melbourne were for initial visits for new referrals in comparison to 6.9% (n = 82) at the SNU in Adelaide.

Appointments were also reviewed in relation to the oral health professional conducting the appointment (Table 1). Although there were comparable numbers of full-time equivalent staff across the two services (RDHM = 6.5 FTE, SNU = 6.2 FTE), there was some variation in terms of workforce personnel, particularly the greater use of dental auxiliaries in Adelaide. Regardless, across both states more than half of all appointments were with general dentists (RDHM: 54.2%, SNU: 54.6%). The second most common oral health practitioner, in terms of appointment numbers, were specialists in special needs dentistry across all clinics except for the Domiciliary service in Victoria where 29.2% of appointments were conducted by dental prosthetists (denture technicians with additional training to provide direct patient care) and more than half (58.1%) by general dentists.

Patient demographics were reviewed for each of the clinics. There was no difference in gender or mean age between those treated in South Australia (55.1 years, σ 15.5) and Victoria (55.3 years, σ 22.0), however, it was clear that older patients were more likely to be treated at certain clinics. In particular, the domiciliary service treated patients that were significantly older than in any other clinic with more than half (57.2%) aged over 75 years (Table 2). Just under one third (n = 207, 31.9%) of referrals to RDHM were for domiciliary care.

**Table 2:** Distribution of age of patients (in years) treated at Royal Dental Hospital of Melbourne (Melbourne, Victoria) and Special Needs Unit (Adelaide, South Australia) clinics categorised by age group [n (%)] and mean age (mean [(95% CI)]

	Royal Dental Hospital of Melbourne			Special Needs Unit, Adelaide			
	ISND	Dom	Total RDHM	ADH	TQEH	Other	Total SNU
< 25	76 (17.3)	2 (0.8)	78 (11.6)	21 (2.1)	6 (3.1)	1 (2.1)	21 (2.1)
25–34	61 (13.9)	3 (1.3)	64 (9.5)	100 (10.1)	9 (4.7)	0 (0.0)	100 (10.1)
35–44	77 (17.5)	5 (2.1)	82 (12.1)	147 (14.8)	7 (3.6)	3 (6.4)	147 (14.8)
45–54	82 (18.7)	25 (10.6)	107 (15.9)	258 (26.0)	37 (19.2)	13 (27.7)	258 (26.0)
55–64	72 (16.4)	29 (12.3)	101 (15.0)	226 (22.8)	36 (18.7)	16 (34.0)	226 (22.8)
65–74	39 (8.9)	37 (15.7)	76 (11.3)	155 (15.6)	42 (21.8)	11 (23.4)	155 (15.6)
75 +	32 (7.3)	135 (57.2)	167 (24.7)	86 (8.7)	56 (29.0)	3 (6.4)	86 (8.7)
Total	439	236	675	993	193	47	1233
Mean (95% CI)	45.6 (43.0–47.3)	73.4 (71.4–75.5)	55.3	53.5 (52.6–54.5)	62.9 (60.5- 65.3)	57.2 (53.0–61.3)	55.1

*ISND* Integrated Special Needs Department and Day Surgery Unit, *Dom* Domiciliary Dental Service, *RDHM* Royal Dental Hospital of Melbourne, *ADH* Adelaide Dental Hospital, *TQEH* The Queen Elizabeth Hospital, *Other* Highgate Park and Modbury GP Plus Superclinic, *SNU* Special Needs Unit

Patients treated were also examined with respect to their eligibility for public dental care, defined as having an Australian government-issued concession health care card or pensioner card. A significantly greater proportion of patients treated by Adelaide-based clinics did not meet eligibility criteria (Table 3). Likewise, the proportion of patients able to self-consent for procedures was significantly greater in South Australian clinics (Table 3). In addition, the ability to self-consent was less likely for patients treated at Highgate Park/Modbury GP Plus Superclinic (n = 1, 2.1%) and amongst those treated by the domiciliary service (n = 76, 32.2%) in comparison to those treated at the ISND (n = 246, 56.0%), Adelaide Dental Hospital (n = 889, 89.5%), or The Queen Elizabeth Hospital (n = 192, 99.5%).

**Table 3:** Eligibility for public dental care and consent status compared between the Royal Dental Hospital of Melbourne (RDHM) in Melbourne, Victoria and Special Needs Unit (SNU) in Adelaide, South Australia

	RDHM [n (%)]	SNU [n (%)]
Eligible	649 (96.1)	1186 (81.1)^a^
Self-consent	322 (48.4)	1082 (89.9)^a^
Family member	321 (48.3)	104 (8.6)
Other mPOA	7 (1.1)	1 (0.1)
Section 42 K	15 (2.3)	2 (0.2)
Other	0 (0.0)	14 (1.2)
Total	665	1203

Other mPOA: Other medical power of attorney, Section 42K: Application process for provision of consent by the Office of Public Advocate in the absence of an identifiable medical treatment decision maker

^a^Statistically significant (p < 0.05)

Patient groups were also compared on the basis of referrals. Where a reason for referral could be identified from the patient records (n = 1830, 95.9%), these were divided into four categories: medical, psychological, disability, and domiciliary care. Patient records where no referral could be identified were excluded from the analysis. ‘Medical’ referrals were defined as those where a medical condition or medication was identified as the primary reason for referral. Referrals that cited anxiety, phobia, or another psychiatric diagnosis likely to impact on dental treatment were categorised as ‘psychological’. Those that identified intellectual or physical disabilities as the primary reason were categorised as’ disability’ referrals.

There were vastly different profiles with regards to referral reason between the two states. Higher rates of psychological and disability-based referrals were seen by ISND in Melbourne whereas the vast majority of patients treated by SNU in Adelaide were for medical reasons (Table 4). When divided by clinic, the South Australian results demonstrated a greater proportion of patients referred for medical reasons were treated at the hospital-based clinics: Adelaide Dental Hospital and The Queen Elizabeth Hospital (Table 4).

**Table 4:** Comparison of reason for referral [n (%)] and medical histories between individual specialist clinics and all appointments with the Royal Dental Hospital of Melbourne (RDHM) in Melbourne, Victoria and Special Needs Unit (SNU) in Adelaide, South Australia

Referral reason	Royal Dental Hospital of Melbourne			Special Needs Unit, Adelaide			
	ISND	Dom	Total RDHM	ADH	TQEH	Other	Total SNU
Medical	112 (27.6)	10 (35.7)	122 (28.1)	781 (80.0)	135 (87.7)	0 (0.0)	916 (77.8)
Psychological	117 (28.8)	6 (21.4)	123 (28.3)	47 (4.8)	17 (11.0)	0 (0.0)	64 (5.4)
Disability	177 (43.6)	12 (42.8)	189 (43.5)	148 (15.2)	2 (1.3)	47 (100.0)	197 (17.6)
Total	406	28	434	976	154	47	1177
No medical history [n (%)]	231 (52.6)	195 (82.6)	426 (63.1)	24 (2.4)	19 (9.8)	11 (23.4)	54 (4.4)
No medication list [n (%)]	80 (18.2)	22 (9.3)	102 (15.1)	3 (0.3)	0 (0.0)	0 (0.0)	3 (0.2)
Number of medical conditions	2.9	3.0	2.9	2.5	3.1	1.9	2.6
95% CI	2.7–3.2	2.4–3.6	2.7–3.1	2.4–2.6	2.8–3.3	1.6–3.3	2.5–2.7
Number of medications	2.3	2.0	2.2	3.6	4.1	2.7	3.6
95% CI	1.9–2.6	1.5–2.5	1.8–2.4	3.4–3.8	3.6–4.5	1.7–3.7	3.5–3.8

*ISND* Integrated Special Needs Department and Day Surgery Unit, *Dom* Domiciliary dental service, *RDHM* Royal Dental Hospital of Melbourne, *ADH* Adelaide Dental Hospital, *TQEH* The Queen Elizabeth Hospital, *Other* Highgate Park and Modbury GP Plus Superclinic

Medical conditions and medications were also reviewed in patient records. Records with no medical history or medication list dated within the 12 months prior to the appointment date were excluded from further analysis (Table 4). When the two states were compared, patients treated in Melbourne had a higher mean number of medical conditions (2.9, σ 1.8 vs. 2.6, σ 11.5; p < 0.05) while those in Adelaide were taking more medications (3.6, σ 3.3 vs. 2.2, σ 3.4; p < 0.05).

A profile of the recorded medical diagnoses of patients treated across the two specialist units, coded and grouped using ICD-10 categories, is provided in Table 5 [13]. All medical diagnoses, not just the primary condition for referral, were included. A much higher proportion of patients treated at SNU in Adelaide had an infectious disease diagnosis or a history of malignant neoplasm. In contrast, there were higher proportions of patients with diseases affecting the nervous system and musculoskeletal system and connective tissues as well as those with mental and behavioural problems, congenital conditions, and sensory disturbances to hearing or sight treated in Melbourne.

**Table 5:** Proportion of patients treated at the Royal Dental Hospital of Melbourne (RDHM) in Melbourne, Victoria and Special Needs Unit (SNU) in Adelaide, South Australia with medical conditions grouped by body system affected

Body system	RDHM (%)	SNU (%)
Infections and parasitic diseases	4.1	33.2
Malignant neoplasms	4.4	24.3
Blood and blood-forming organs	5.8	4.1
Endocrine, nutritional, and metabolic	27.1	22.9
Mental and behavioural	61.8	25.8
Nervous system	28.2	13.3
Eye and ear	3.9	0.6
Circulatory system	34.9	36.0
Respiratory system	15.1	18.0
Digestive system	17.0	12.9
Skin and subcutaneous tissue	2.3	2.0
Musculoskeletal system and connective tissue	25.0	14.1
Genitourinary system	4.8	4.7
Congenital abnormalities and conditions originating in the perinatal period	9.0	3.9
Physical or brain injuries	2.7	2.1

More than one medical condition could be coded per patient

Dental treatment received at appointments was also compared between clinics. Treatment was categorised into six groups (Diagnostic, Periodontics, Restorative, Endodontics, Oral surgery, Other) based on the item numbers recorded for the appointment. The Other category primarily consisted of treatments involved in denture fabrication. Treatment did not differ significantly between the Melbourne and Adelaide clinics overall although a greater proportion of treatments in the Oral Surgery and Other categorises were provided at the ISND (Melbourne, Victoria). The frequency of endodontic procedures at SNU (Adelaide, South Australia) was double that completed at ISND (Melbourne, Victoria). The number of patients that failed to attend their dental appointment was higher in Adelaide with the number of failed appointments at the Adelaide Dental Hospital (33.6%) more than double that of the ISND (Melbourne, Victoria) (14.4) (Table 6).

**Table 6:** Dental treatments completed at appointments [n (%)] categorised by specialist dental clinic and overall for the Royal Dental Hospital of Melbourne (RDHM) in Melbourne, Victoria and Special Needs Unit (SNU) in Adelaide, South Australia

	Royal Dental Hospital of Melbourne			Special Needs Unit, Adelaide			
	ISND	Dom	Total RDHM	ADH	TQEH	Other	Total SNU
Diagnostic	186 (49.5)	89 (45.4)	275 (48.1)	393 (57.0)	60 (34.5)	32 (68.1)	485 (53.3)
Periodontics	73 (19.4)	17 (8.7)	90 (15.7)	101 (14.7)	7 (4.0)	26 (55.3)	134 (14.7)
Restorative	69 (18.4)	12 (6.1)	81 (14.1)	138 (20.0)	44 (25.3)	8 (17.0)	190 (20.9)
Endodontics	7 (3.6)	0 (0.0)	7 (1.2)	9 (1.3)	18 (10.3)	0 (0.0)	27 (3.0)
Oral surgery	33 (8.8)	27 (13.8)	60 (10.5)	10 (1.5)	16 (9.2)	0 (0.0)	26 (2.9)
Other	39 (10.4)	54 (27.6)	93 (16.3)	2 (0.3)	1 (0.6)	0 (0.0)	3 (0.3)
FTA	63 (14.4)	40 (16.7)	103 (15.3)	304 (33.6)	19 (9.8)	0 (0.0)	323 (26.2)

*ISND* Integrated Special Needs Department and Day Surgery Unit, *Dom* Domiciliary service, *RDHM* Royal Dental Hospital of Melbourne, *ADH* Adelaide Dental Hospital, *TQEH* The Queen Elizabeth Hospital, *Other* Highgate Park and Modbury GP Plus Superclinic, *SNU* Special Needs Unit, *FTA* Failed to attend appointment

## Discussion

Countries have sought to address issues relating to access to care for individuals with special needs in different ways with Australia and New Zealand leading many parts of the world in recognising special needs dentistry as a dental specialty [7]. However, despite a number of specialised clinics having been established across Australia over the last 15 years, little is known about the utilisation of these services.

The results of this cross-sectional audit of two of Australia’s largest and most established referral centres for the dental management of individuals with special needs; the Integrated Special Needs Department (ISND) at the Royal Dental Hospital of Melbourne (RDHM) in Melbourne, Victoria and the Special Needs Unit (SNU) in Adelaide, South Australia, provide an initial understanding of the profile of patients referred for specialist care and the treatment they receive. Despite the expected homogeneity of the populations serviced by these clinics, there were, in fact, differences when the profile of patients was compared. In particular, patients treated in Melbourne were more likely to have been referred because of either a physical or intellectual disability and were generally from older age groups. In contrast, those treated in Adelaide were more likely to be referred because of the impact of a medical condition on their oral health or dental treatment with a greater proportion not meeting conventional eligibility criteria for public dental care in Australia.

The difference in reason for referral between the two centres was of particular interest. The existing literature regarding referrals of patients with special needs in Australia is limited. A previous audit of referrals to RDHM (Melbourne, Victoria) found that the majority of patients (81.7%) were referred for medical reasons with a much smaller proportion due to behavioural issues (14.0%) or intellectual disability (21.0%) [10]. The contrast with the results presented here is a consequence of differences in methodology with Rohani et al. allowing for patients to be referred for multiple reasons [10]. The results of our study were consistent with the more common findings in the literature that general dentists tend to feel least comfortable with treating individuals with intellectual disabilities or behavioural problems and are thus more likely to refer them for specialist care [2–5].

The higher proportion of disability patients managed by the Melbourne unit may be the result of a number of reasons the first of which may be the influence of services offered, particularly the internal access to general anaesthetic lists at RDHM in comparison to Adelaide. Given the benefit of this modality in assisting in the provision of dental care to those who may not be able to accept conventional dental treatment, commonly those behavioural and compliance issues as has been described in the literature, this may be unsurprising compared to the proportion in the Adelaide clinic [15].

In a similar manner, the results demonstrate clearly how the domiciliary service in Melbourne improves access to dental care for older members of the community who may find it difficult attending a dental clinic. In the context of an aging population, and particularly for dependent older adults in residential aged care facilities, such modalities are important ways in which health care provision can be adapted to improve access to these individuals who experience poorer oral health and greater unmet treatment need [16–19].

The second reason for the lower proportion of disability patients at the Adelaide clinic is likely to be a reflection of the concerted efforts made by the unit to support clinicians in the community dental clinics through its ‘Special Needs Network’. This initiative allows specialists to work in close collaboration with upskilled dentists in the community allowing patients with disabilities to be able to access care closer to home and for only more complex cases to be referred to specialists either at the Adelaide Dental Hospital or outreach clinics such as Modbury GP Plus Superclinic.

In a similar manner, the higher proportion of patients with complex medical issues treated at SNU in Adelaide may also be a result of the proximity of the Adelaide Dental Hospital to the Royal Adelaide Hospital. Although the RDHM sits close to Melbourne’s biomedical precinct, several of Melbourne’s tertiary- and quaternary-referral hospitals have their own dedicated dental clinics reducing the need to refer to external facilities.

The medical profile of patients treated at the SNU in Adelaide is relatively characteristic of patients referred for specialist dental care in Australia and internationally [11, 12, 20, 21]. In addition, the number and range of comorbid medical conditions and medications across the two centres provides an indication of the complexity of managing the oral health care needs of these individuals in the context of their overall health status.

Of particular interest and concern are the almost 20% of patients treated at the Adelaide clinic who do not meet conventional eligibility criteria for public dental care with similar proportions reported at other hospital-based clinics in Australia [11, 12]. In many cases, these individuals have access to a course of specialist dental treatment in the public dental systems in preparation for significant medical interventions but do not receive ongoing care as they do not meet financial disadvantage criteria. The chronicity and long-term side effects of many of these medical conditions and interventions, such as is the case with head and neck cancer patients, place these individuals at increased risk of dental disease and raises the question of whether these individuals are able to access appropriate and necessary dental care in the private dental sector where specialist care of this nature is limited. The anecdotal experience of specialists has been that many are referred back many years later with significant deterioration of their oral health despite care from their general dental practitioners.

The results of this study also reported for the first time on the nature of treatments completed at specialty clinics for those with special needs in Australia. Overall, although the nature of treatment provided between the two units was similar there was some variation in the procedures completed at the hospital-based clinics potentially reflecting differences in the relationships between specialty units at the respective facilities or the reluctance of other specialists to manage individuals with disabilities as has been discussed in the literature [22]. The findings reinforce the need for clinicians working with individuals with special needs to be highly skilled across all areas of dentistry to be able to meet the treatment needs of their patients.

An interesting finding was the difference in number of appointments and patients seen between the two services despite having comparable numbers of full-time equivalent staff. These findings are somewhat a reflection of the nature of services provided at each clinic. For example, less patients are treated on general anaesthetic lists due to higher number of treatments completed in a single session and domiciliary visits require allowances for travel between patients. These factors need to be considered in funding models for dental services as conventional metrics of patient numbers and treatment codes may not reflect the additional time and complexity of managing patients with special needs.

Similarly, these differences also highlight the importance of effective use of the workforce in managing demand for services for individuals with special needs. The Adelaide clinic used a model whereby different clinicians managed the treatment for the same patient. This allowed specialists to focus on the needs of more complex patients while being supported by general dentists, oral health therapists, and dental hygienists in their relative areas of experience and scope of practice. In contrast, patients in Melbourne generally saw a single clinician for the entirety of their care. The merits of each model should be considered in the context of how access to care is impacted by the limited number of clinicians willing to treat patients with special needs but also what may be beneficial based on individual patient needs.

In addition, another interesting finding was that there was a significantly higher number of patients that failed to attend appointments at the Adelaide clinic. The existing literature suggests that non-attendance at appointments is more likely to be associated with socioeconomic disadvantage with reasons often provided including illness, dental anxiety, forgetfulness, and a lack of understanding about the importance of ongoing care [23–25]. Given the degree of medical complexity of patients treated at the SNU, many of these could be viable explanations given a larger proportion of appointments were for ongoing care in comparison to more appointments being for new referrals in Melbourne. Regardless, further investigations are required to ensure attendance at this clinic was not reflective of other barriers to accessing care, such as problems with transportation, that have been proposed for individuals with special needs [4]. There were, however, deficiencies in the current study. These largely pertained to the period of the clinical audit being limited to one month and the restriction to only two referral centres due to other centres declining to be involved in this study. Another was the relatively high rates of incomplete medical histories within the patient records reviewed. Although this may have influenced the medical profile of patients in the current study, of greater concern was the possible impact on safety of patient care given the complexity of individuals with special needs and how vital a knowledge of their medical issues can be to their oral health and treatment provision [26].

The lack of compliance in taking medical histories potentially echoes concerns from within the special needs community that oral health professionals continue to have inadequate training and experience in managing individuals with special needs. The results of this study provide an interesting initial profile of the types of patients referred to two of the largest and most well-established specialist units in Australia for treatment and reflect, to a certain extent, how the wider dental profession defines an individual with ‘special needs’ requiring specialist level care. It reinforces the vital and important work completed by the limited specialist workforce and some of the challenges they face in meeting the health care demands of the growing group of individuals in the population with additional health care needs. Ultimately, reflecting on the profile of patients referred for specialist care in Australia should prompt other countries to consider whether their health care systems are currently meeting the needs of these individuals or that establishing a specialty in special needs dentistry is certainly part of the solution to address issues relating to access to care for the growing number of individuals with special needs in our community.

## Conclusions

This study provides an initial profile of patients with special needs referred for specialist care at two of Australia’s largest referral centres: the Integrated Special Needs Department in Melbourne and the Special Needs Unit in Adelaide. In addition to highlighting the medical complexity of this group of patients, the results also suggest that service provision models and workforce may influence referrals and access to care, particularly the use of domiciliary services for functionally-dependent older adults. Differences between the two centres raise the need to consider if, in fact, these reflect potential barriers to access of care for patients with special needs. Regardless, this study demonstrates the important role this specialty plays in managing the oral health needs of this complex group of patients.

## Data Availability

The datasets used and analysed during this study are available from the corresponding author on reasonable request.

## Abbreviations

ADH: Adelaide Dental Hospital
ISND: Integrated Special Needs Department
RDHM: Royal Dental Hospital of Melbourne
SNU: Special Needs Unit
TQEH: The Queen Elizabeth Hospital

## References

[1] Nelson LP, Getzin A, Graham D, Zhou J, Wagle EM, McQuiston J (2011). Unmet dental needs and barriers to care for children with significant special health care needs. Pediatr Dent.

[2] Pradhan A, Spencer A, Slade G (2007). Factors influencing oral health of adults with physical and intellectual disabilities in various living arrangements. Aust Dental J.

[3] Pradhan A, Slade GD, Spencer AJ (2009). Factors influencing caries experience among adults with physical and intellectual disabilities. Commun Dent Oral Epidemiol.

[4] Pradhan A, Slade GD, Spencer AJ (2009). Access to dental care among adults with physical and intellectual disabilities: residence factors. Aust Dent J.

[5] Derbi HA, Borromeo GL (2016). The perception of Special Needs Dentistry amongst general dentists within Western Australia, Australia. J Gerontol Geriatric Res.

[6] New Zealand Dental Association. Specialisation 2013 [cited 2015 2 May]. https://www.nzda.org.nz/pub/index.php?id=337&no_cache=1.

[7] Dental Board of Australia. List of specialties. www.dentalboard.gov.au/.../default.aspx?record=WD10%2F3238&dbid=AP&chksum=hXwmbYjUdcXv23v2lFcC3w%3D%3D.

[8] Borromeo G, Bramante G, Betar D, Bhikha C, Cai Y, Cajili C (2014). Transitioning of special needs paediatric patients to adult special needs dental services. Aust Dent J.

[9] Royal Australasian College of Dental Surgeons. Specialist Dental Practice 2014 [cited 2015 2 May]. http://www.racds.org/RACDSNEW_Content/Education/Specialist_Dental_Practice.aspx.

[10] Rohani M, Calache H, Borromeo M (2017). Referral patterns of special needs patients at the Royal Dental Hospital of Melbourne, Victoria, Australia. Aust Dent J.

[11] Lim MAWT, Borromeo GL (2017). Patient referrals to special needs dental units in Tasmania, Australia. J Disabil Oral Health.

[12] Lim MAWT, Borromeo GL (2017). Special Needs Dentistry: Interdisciplinary management of medically-complex patients at hospital-based dental units in Tasmania, Australia. Int J Med Res Health Sci.

[13] World Health Organization. ICD-10 Version: 2016: World Health Organization; 2016 [cited 2016 5 June]. http://apps.who.int/classifications/icd10/browse/2016/en.

[14] World Health Organization Collaborating Centre for Drug Statistics Methodology. ATC/DDD Index 2016 Oslo, Norway: Norwegian Institute of Public Health; 2016 [updated 16 December 2015; cited 2016 5 June]. http://www.whocc.no/atc_ddd_index/.

[15] Lim MAWT, Borromeo GL (2017). The use of general anesthesia to facilitate dental treatment in adult patients with special needs. J Dental Anesth Pain Med.

[16] Fiske J, Lewis D (2000). The development of standards for domiciliary dental care services: guidelines and recommendations. Gerodontology.

[17] Silva M, Hopcraft M, Morgan M (2014). Dental caries in Victorian nursing homes. Aust Dent J.

[18] Chalmers J, Carter K, Fuss J, Spencer A, Hodge C (2002). Caries experience in existing and new nursing home residents in Adelaide. Aust Gerodontol.

[19] Chalmers JM (2003). Oral health promotion for our ageing Australian population. Aust Dent J.

[20] Monteserín-Matesanz M, Esparza-Gómez GC, García-Chías B, Gasco-García C, Cerero-Lapiedra R (2015). Descriptive study of the patients treated at the clinic “Integrated Dentistry for Patients with Special Needs” at Complutense University of Madrid (2003–2012). Med Oral Patol Oral Cirugia Bucal.

[21] Ahmad MS, Shafie NE, Redhuan TM, Mokhtar IW (2019). Referral pattern and treatment needs of patients managed at a Malaysian special care dentistry clinic. J Int Oral Health.

[22] Yap E, Parashos P, Borromeo G (2015). Root canal treatment and special needs patients. Int Endod J.

[23] Listl S, Moeller J, Manski R (2014). A multi-country comparison of reasons for dental non-attendance. Eur J Oral Sci.

[24] Herrick J, Gilhooly MLM, Geddes DAM (1994). Non-attendance at periodontal clinics: forgetting and administrative failure. J Dent.

[25] Collins J, Santamaria N, Clayton L (2003). Why outpatients fail to attend their scheduled appointments: a prospective comparison of differences between attenders and non-attenders. Aust Health Rev.

[26] De Angelis A, Chambers I, Hall G (2010). The accuracy of medical history information in referral letters. Aust Dent J.

